# Rapid identification of *Brucella *isolates to the species level by real time PCR based single nucleotide polymorphism (SNP) analysis

**DOI:** 10.1186/1471-2180-8-86

**Published:** 2008-06-02

**Authors:** Krishna K Gopaul, Mark S Koylass, Catherine J Smith, Adrian M Whatmore

**Affiliations:** 1 Division of Statutory and Exotic Bacteria, Veterinary Laboratories Agency, Addlestone, UK

## Abstract

**Background:**

Brucellosis, caused by members of the genus *Brucella*, remains one of the world's major zoonotic diseases. Six species have classically been recognised within the family *Brucella *largely based on a combination of classical microbiology and host specificity, although more recently additional isolations of novel *Brucella *have been reported from various marine mammals and voles. Classical identification to species level is based on a biotyping approach that is lengthy, requires extensive and hazardous culturing and can be difficult to interpret. Here we describe a simple and rapid approach to identification of *Brucella *isolates to the species level based on real-time PCR analysis of species-specific single nucleotide polymorphisms (SNPs) that were identified following a robust and extensive phylogenetic analysis of the genus.

**Results:**

Seven pairs of short sequence Minor Groove Binding (MGB) probes were designed corresponding to SNPs shown to possess an allele specific for each of the six classical *Brucella *spp and the marine mammal *Brucella*. Assays were optimised to identical reaction parameters in order to give a multiple outcome assay that can differentiate all the classical species and *Brucella *isolated from marine mammals. The scope of the assay was confirmed by testing of over 300 isolates of *Brucella*, all of which typed as predicted when compared to other phenotypic and genotypic approaches. The assay is sensitive being capable of detecting and differentiating down to 15 genome equivalents. We further describe the design and testing of assays based on three additional SNPs located within the 16S rRNA gene that ensure positive discrimination of *Brucella *from close phylogenetic relatives on the same platform.

**Conclusion:**

The multiple-outcome assay described represents a new tool for the rapid, simple and unambiguous characterisation of *Brucella *to the species level. Furthermore, being based on a robust phylogenetic framework, the assay provides a platform that can readily be extended in the future to incorporate newly identified *Brucella *groups, to further type at the subspecies level, or to include markers for additional useful characteristics.

## Background

Brucellosis remains one of the world's major zoonotic disease problems and can lead to reproductive problems in a number of large livestock and other animals. In humans, brucellosis is associated with a broad spectrum of symptoms and can occasionally be fatal [1]. Whilst brucellosis is the global term for infection by organisms in the Gram-negative genus *Brucella*, there are actually a number of causative agents. Differentiation to species level within the genus is based on both different phenotypic characteristics and the host from which it was initially isolated [2]. These divisions are also supported by pulse field gel electrophoresis (PFGE)-based physical mapping, showing that each species has a specific physical organisation [3], and techniques such as multilocus sequencing [4] and variable number tandem repeat (VNTR) based typing [5,6]. Bovine infection is generally caused by *B. abortus*, *B. melitensis *infects predominantly sheep and goats, *B. suis *infects pigs, hares and reindeer, *B. ovis *infects sheep only, *B. canis *infects dogs, whilst *B. neotomae *has only been isolated from the desert wood rat. In addition to the six classically described species novel members of the genus have more recently been isolated from marine mammals, predominantly dolphins, porpoises and seals. These have very recently been classified as two additional species, *B. ceti *(cetaceans) and *B. pinnipedialis *(pinnipeds) [7]. In addition, a novel species of *Brucella *(named as *Brucella microti*), associated with voles, has recently been reported [8,9]. Some *Brucella *species are further sub-divided into groups known as biovars. Whilst these are of epidemiological interest in *B. abortus *and *B. melitensis*, the five biovars that are found in *B. suis *appear to relate to host preference. In humans, four of the classical species (not *B. ovis *or *B. neotomae*) have been shown to cause disease [2]. There is also evidence to suggest that humans can be infected by marine mammal *Brucella *further highlighting the importance of this group of organisms to human health [10-12].

Identification to species level is traditionally based on biotyping that examines antigenic, phenotypic and phage susceptibility profiles. While the approach has been useful for many years, it has a number of recognised drawbacks. Being culture based, biotyping requires specialist handling facilities for the initial isolation of a pure culture and then needs additional culture time for phenotype and phage susceptibility determination. In addition, some of the differentiating characteristics can be very subtle meaning that biotyping should only be carried out by highly experienced individuals. However, even with this caveat, interpretation of this method is acknowledged to be rather subjective. Furthermore there are frequent reports of atypical organisms that do not conform to the expected characteristics of particular species.

Molecular biology provides tools that can circumvent such problems associated with classical microbiology by using suitably validated genetic markers for objective and unambiguous identification of groups. As DNA is the target of this testing, culturing can be reduced or potentially bypassed, both removing the necessity for specialist handling facilities, and shortening time from sampling to identification. With the explosion of molecular sequence data and an expanding array of technological platforms the use of assays based on single nucleotide polymorphisms (SNPs) in microbial genotyping is becoming increasingly common. For example, point mutations in *rpo*B and *kat*G have been used as markers for resistance to rifampicin and isoniazid in *Mycobacterium tuberculosis *[13] while SNPs have also been used to differentiate vaccine and field strains of canine parvovirus [14]. In terms of microbial diagnostics SNP-based genotyping has been applied to a number of organisms including members of the *Mycobacterium tuberculosis *complex [15], *Bacillus anthracis *[16] and *Burkholderia *species. [17].

We have recently undertaken extensive phylogenetic analysis of the *Brucella *group involving the sequencing of some 21 distinct gene fragments, from over 400 strains of *Brucella*, representing the known genetic diversity of the group [4, M.S. Koylass and A.M. Whatmore, unpublished data]. While this extensive analysis confirmed the long-standing view that the genus is highly homogeneous [18-20] it has nevertheless allowed us to confidently identify SNPs that we believe define each of the classically accepted *Brucella *species. Furthermore SNPs are particularly powerful markers in such a conserved group as they are highly unlikely to have occurred twice independently (i.e. in distinct evolutionary branches) and equally, having occurred, are unlikely to mutate again back to their ancestral state [21]. In addition, these studies revealed strong linkage disequilibrium in the distribution of alleles between *Brucella *species [4] implying that recombination is rare and giving increased confidence in the robustness and stability of species-specific SNPs selected as markers.

We have thus selected seven SNPs that we have worked into a multiple outcome assay to identify *Brucella *isolates at the species level. An assay based on these markers should deliver unambiguous results without the subjectivity associated with biotyping or problems associated with strains that are phenotypically aberrant. We recently described the development of a SNP-based assay identifying *Brucella *to the species level on a primer extension platform [22]. Here, we describe the development of an equivalent tool for the real-time PCR platform that has certain advantages in terms of speed, technical simplicity and potentially wider applicability being based on a cheaper, and more widely available, technological platform. We describe the use of a panel of short Taqman 5' labelled, 3' Minor Groove Binding (MGB), probes to interrogate species defining SNPs. Use of the MGB protein raises the melting temperature of probes meaning that a single base mismatch causes more destabilisation than would be the case with a longer probe [23]. This facilitates accurate SNP detection allowing us to use this approach in an assay to identify *Brucella *to the species level.

## Results

### Assay design

Previous multilocus sequence analysis (MLSA) studies, performed by us, identified a number of SNPs that are specific for known *Brucella *spp. These form the basis of the assay described here (see Table 1). Point changes specific for *B. melitensis*, *B. ovis*, *B. canis *and the marine mammal Brucella were described previously [22] based on multi-locus sequence analysis of 160 strains. The SNPs specific for *B. abortus*, *B. suis*, and *B. neotomae *used in this assay, were identified by extension of this work to examine a total of 21 gene fragments from over 400 strains (M. Koylass and A. M. Whatmore, unpublished data). In the case of *B. suis *it was not possible to identify a single SNP specific to the classical taxonomic group. This reflects the fact that *B. suis *biovar (bv) 5 is genetically distinct from the remaining *B. suis *biovars [4,6,24]. The SNP used in the assay described here therefore identifies only *B. suis *bvs 1–4. There are SNPs specific to *B. suis *bv 5 [4] that could be used in an assay to discriminate this biovar, although in practical terms it has only rarely been isolated from rodents and is of no diagnostic significance. The *B. suis *bvs 1–4 SNP in *prp*E is shared with *B. canis *reflecting the fact that *B. canis *is embedded within the *B. suis *phylogenetic cluster. However the presence of a SNP confined to *B. canis *in *omp*25 allows unambiguous separation of these two species [4]. In previous work, where we used primer extension to determine species, we had not incorporated a target to positively identify *B. suis *but rather used a process of elimination to identify members of this species. However, with the isolation of novel species such as *B. microti*, it became necessary to redress this problem. Brucellae isolated from marine mammals have recently been formally designated as two distinct species, *B. pinnipedialis *and *B*. *ceti *[7]. However, this designation is proving controversial as it is inconsistent with apparent genetic divisions within the marine mammal group [25,26]. Reflecting this, it has not been possible to identify SNPs that specifically identify these two species. Thus the SNP chosen for use in this assay, in *trp*E, is universal to all marine mammal isolates [4].

**Table 1:** Gene targets, primers and allele-specific probes used in this study.

Species	Gene target	Location in *B. abortus *9–941	Primers		Probes	
*B. abortus*	*fba*A	AE017224	F	TGACATCATGCTCCGTCACATG	VIC	ATGCCGTGGCGGAA
		360225–361289	R	CAGACCGGAATATGCGGATAGAT	FAM	ATGCCGTGACGGAA
*B. melitensis*	*gap*	AE017223	F	GGCTCAGGTTCTCAACGATACTATC	VIC	CGTGGTCATAAAGC
		1684721–1685728	R	TCGCCCGTATAGGAGTGGAT	FAM	CGTGGTCATGAAGC
*B. ovis*	*aro*A	AE017223	F	CGACCACCGCATCGC	VIC	CCATGACAAGGAAAC
		29246–30598	R	CCGGCTTTTCCGATGCAA	FAM	CATGACGAGGAAAC
*B. suis *bv1–4	*prpE*	AE017223	F	GCGACCGCATCCTCATCTATATG	VIC	CAAGCGTGGCAACC
		1687718–1689625	R	CGCCGAATACGACGGAATGAAT	FAM	CAAGCATGGCAACC
Marine mammal Brucellae	*trp*E	AE017223	F	CGAGGATTCCTTCGTCCATACG	VIC	CCAATTATTTCCACCAGACG
		1537355–1539550	R	ACGCACGGTGGAAACCTT	FAM	CCAATTATTTCCGCCAGACG
*B. canis*	*omp*25	AE017223	F	GCTGGCGCCTTTGCT	VIC	AACTTCCAGAAGGACC
		710024–710625	R	GGCCGTCCTTGGACTTCTTG	FAM	AACTTCCAGCAGGACC
*B. neotomae*	putative oxido-reductase	AE017223	F	GGTTTTCCATGCGGTTTATTTGC	VIC	CATTGAGTGGCCCGAT
		1989869–1990870	R	GGCATCATGCACAGTGATATCGA	FAM	ATTGAGCGGCCCGAT
16SrRNA_771/778_	16SrRNA	-	F	CGCCGTAAACGATGAATGTTAGC	VIC	CGAAGTGTAAACACCCCGA
			R	GCGGAATGTTTAATGCGTTAGCT	FAM	CGAAGAGTAAACTCCCCGA
16SrRNA_1055_	16SrRNA	-	F	GGGTTAAGTCCCGCAACGA	VIC	ACCCTCGCCTTTAGTT
			R	ACGTCATCCCCACCTTCCT	FAM	CCCTCGCCCTTAGTT

Gene targets, primers and allele-specific probes used in this study.  *Brucella *species-specific SNPs and non-specific SNPs (both underlined)  are shown within the relevant probes. Gene target locations are given  relative to the sequenced *B. abortus* 9-941 strain.

Individual assays for each SNP are based on the use of two alternative probes, one that preferentially binds to the species-specific polymorphism, while the other binds to the alternative allele state present in members of all other species. In each case the species-specific probe is labelled with VIC while the alternative state probe is labelled with FAM (see Table 1). By following a strategy of optimising each SNP discrimination assay to identical cycling conditions all seven species-defining assays can be analysed concurrently in a multiple outcome assay.

### Optimisation

The single-tube assay, as provided by Applied Biosystems, is provided with a standard probe and primer concentration. However upon initial testing, certain assays displayed background with some probes not predicted to react in a particular sample, giving fluorescence values above those expected. To overcome this we obtained individual assay components and optimised primer and probe concentrations to maximise discrimination (data not shown). Details of final probe and primer concentrations selected based on this work are presented in Table 2.

**Table 2:** Optimised primer and probe concentrations determined for the seven species defining MGB assays.

**MGB assay**	**Final Forward primer concentration (nM)**	**Final Reverse primer concentration (nM)**	**Final VIC labelled probe concentration (nM)**	**Final FAM labelled probe concentration (nM)**
*B. abortus*	500	700	300	100
*B. melitensis*	700	1100	200	100
*B. ovis*	300	300	250	50
*B. suis *bv1–4	300	300	200	100
*B. canis*	700	1100	250	200
Marine mammal *Brucella*	500	1100	300	50
*B. neotomae*	500	700	200	100

Primer and probe concentrations were optimised to give the greatest amount of discrimination with the least background using the minimum amounts of the various components.

### Assay performance

**Figure 1:**
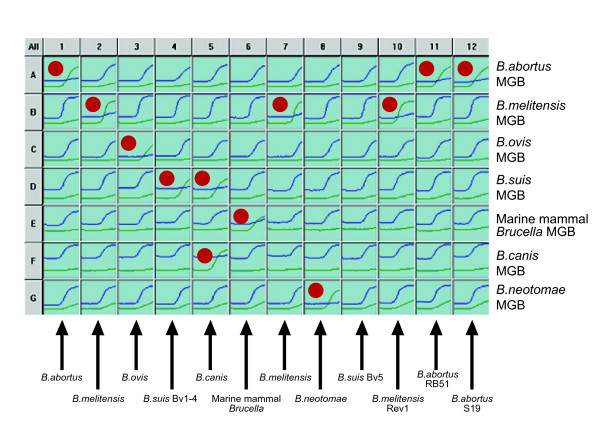
**Seven Species defining MGB assay profile**. Example of the multiple outcome assay used to identify twelve *Brucella *isolates. Each species-defining assay is run in rows (A-G) with samples run in columns. The green PCR profiles represent reactions with the VIC labelled probe, representing the species-specific probe in each probe pair. The blue PCR profiles represent reactions with the FAM labelled probes, representing the reaction with the alternate state (non species-specific) allele probe in each probe pair. The identity of each of the isolates 1–12 is indicated by a red dot where an isolate generates a positive PCR reaction with a VIC-labelled probe.

The optimised assay was run as shown in Figure 1 with each individual SNP discrimination assay running in rows (e.g. A1–A12 = *B. abortus*, B1–B12 = *B. melitensis etc*.). This enables typing of twelve isolates on a single 96-well plate. Reaction plots with the species-specific VIC labelled probes are in green, with the alternative FAM labelled probe in blue. In most cases each sample should generate a strong curve with the VIC labelled probe in only one well, and should react preferentially with the FAM-labelled probe in the remaining six wells. In this manner the sample in column one is identified as *B. abortus*, that in column 2 as *B. melitensis *and so on. Reflecting their phylogenetic position described above, *B. canis *isolates react with the VIC probes for both *B. suis *and *B. canis *(see column 5). The isolate in column 9 is *B. suis *bv 5 which as described above lacks any of the species defining SNPs and thus reacts only with all seven FAM-labelled alternative state probes. This is also the reaction outcome expected with any novel isolates that are not members of classically recognised species or the marine mammal *Brucella *groups.

### Assay validation

**Figure 2:**
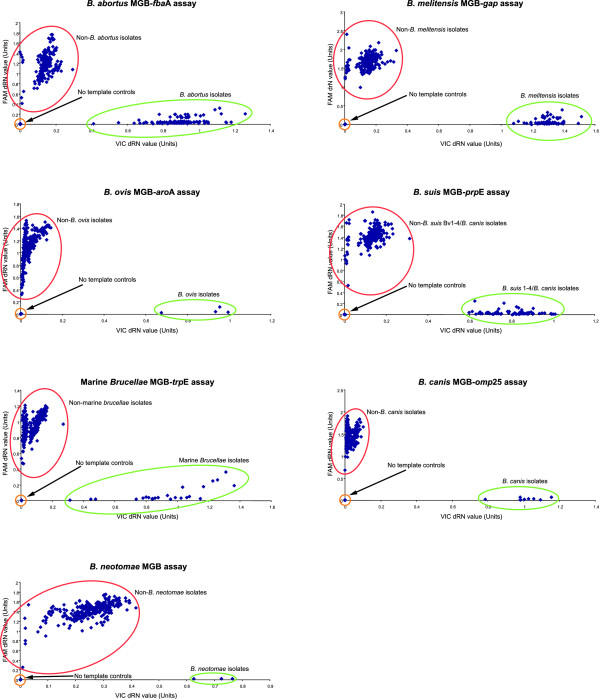
**Discrimination of the seven species defining assays**. Allele discrimination plots generated by each species-defining MGB assay when applied to 303 *Brucella *isolates. Each assay was read after 40 cycles.

Assay validation was performed using 303 *Brucella *isolates encompassing all species and biovar reference strains and a collection of geographically and temporally distinct field isolates comprising members of all known species (Table 3). Alongside representatives of all species and biovars, the vaccine strains *B. abortus *S19 and RB51 as well as *B. melitensis *Rev1 were also included. In addition, strains that had both genotypes and phenotypes that did not correspond to any of the known classical *Brucella *species but were shown to be *Brucella *by 16S rRNA typing were also tested. When all seven individual assays were compared the predicted species identity of the 303 strains was consistent with identification based on other molecular and phenotypic methods. Based on this analysis allele discrimination plots were generated for each SNP assay (Figure 2). Each data-point represents the normalised end-point fluorescence reading for both VIC and FAM labelled probes plotted on the X-axis and Y-axis respectively. Each plot shows that there is unambiguous discrimination between genotypes with two distinct populations. One population clusters around the X-axis and consists of isolates that would be classified as a member of the species targeted by this one specific SNP. The second population clustering around the Y-axis represents members of the remaining species. No template controls cluster around the plot origin with negligible reaction with either probe. The spread of data-points in the scatter plot reflects the fact that DNA concentrations applied to the assay were not normalised.

**Table 3:** Phenotype based identity of the 303 isolates used to validate the species defining MGB assay.

**Species/Biovar**	**Numbers present**
*B. abortus *unknown biovar	22
*B. abortus *bv1	55
*B. abortus *bv2	6
*B. abortus *bv3	11
*B. abortus *bv4	8
*B. abortus *bv5	4
*B. abortus *bv6	11
*B. abortus *bv7	5
*B. abortus *bv9	1
*B. melitensis *unknown biovar	23
*B. melitensis *bv1	7
*B. melitensis *bv2	2
*B. melitensis *bv3	32
*B. ovis*	5
*B. suis *unknown biovar	30
*B. suis *bv1	13
*B. suis *bv2	8
*B. suis *bv3	3
*B. suis *bv4	5
*B. suis *bv5	2
Marine Mammal Brucellae	30
*B. canis*	12
*B. neotomae*	3
Novel phenotype/*Brucella *by 16S rRNA	5

A number of isolates that represent likely new *Brucella *groups, based on 16S rRNA sequencing and other extensive analysis, were tested in the assay including recently described *B. microti *[8,9] and an atypical isolate from a human infection [27]. As with *B. suis *bv 5, seven non-specific curves were generated showing that this assay would identify isolates such as these as *Brucella*, but recognise that they did not belong to any of the currently described species. Within the 303 isolates tested were three reference strains (one of *B. abortus, B. melitensis *and *B. suis*) each cultured *in vitro *through up to 35 passages. Testing of these isolates showed that the target SNPs were stable even on extensive *in vitro *culture.

### Limits of detection of genotyping assay

**Figure 3:**
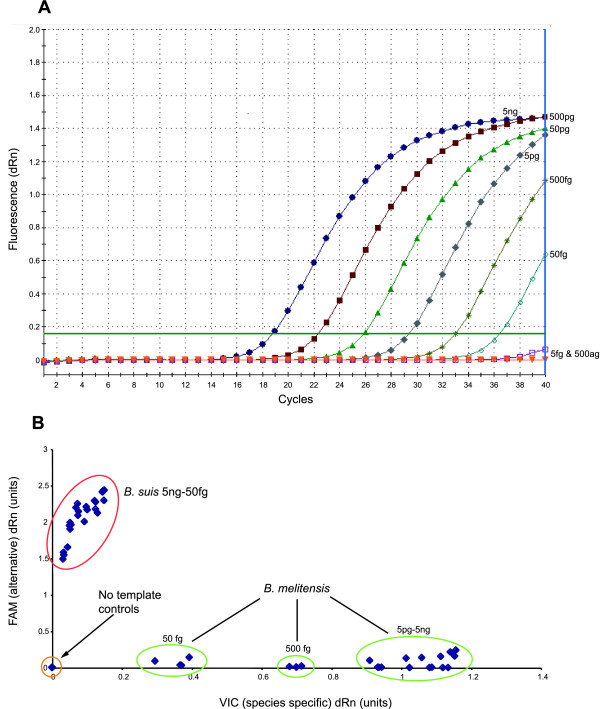
**Sensitivity and limits of discrimination using MGB assay**. Sensitivity of the species defining assays. The data presented here relates to the *B. melitensis gap *assay with the remaining six assays giving equivalent results. A. Titrations of *B. melitensis *16 M DNA from 5 ng to 500 ag. B. Allele discrimination plot showing performance of the *B. melitensis *assay in distinguishing *B. melitensis *from non-*B. melitensis *(*B. suis*) down to 50 fg of DNA.

The analytical sensitivity of each MGB dual probe assay was firstly tested at least four times for each serial dilution from 5 ng (1.5 × 10^6 ^genome equivalents) to 0.5 fg (1.5 × 10^-1 ^genome equivalents) of its target species/type. A representative example is shown in Figure 3a. Each individual assay could reliably detect DNA down to 50 fg, or approximately 15 cells from all the assays. Samples containing 5 fg could only be detected sporadically. Analytical sensitivity was in line with work done on *Bacillus anthracis*, where the lowest detection levels were 100 fg or 17 cells [16]. Allelic discrimination plots for each individual SNP assay comparing serial dilutions of target and non-target species illustrated that there was still clear discrimination even at 50 fg of DNA (for example see Figure 3b).

### Specificity

Initial optimisation work was carried out using the Taqman Universal mix which was effective for all seven assays although fluorescence levels were lower for the *B. abortus *probe pair. This led to lower sensitivity at 40 cycles for this probe pair, although allelic discrimination was unaffected. In order to check specificity of the assay five type strains of *Ochrobactrum*, the closest phylogenetic neighbour of *Brucella *[28], were tested in duplicate. They were shown not to give fluorescence readings greater than the background with all seven probe pairs, which is the very least we would see if these samples were *Brucella*. However, to improve upon the low fluorescence and sensitivity associated with the *B. abortus *probes, we switched to an improved reaction mix (TaqMan Genotyping mix) and then repeated the work. Upon repetition of the *Ochrobactrum *work with the new mix, it was noted that we were now seeing a seven probe profile for the five species that had not been seen previously. However, the kinetics of these reactions were clearly very different to those seen with comparable concentrations of *Brucella *DNA. Reactions were weak with very low endpoint fluorescence values (data not shown). When the targets of SNP assay were compared against the published *O*. *anthropi *ATCC49188 genome sequence, it was found that there were polymorphisms in most of the primers and probes used in this assay that might account for these differences in kinetics (data not shown). This assay was not designed as a diagnostic assay to distinguish *Brucella *from other bacteria, and there are other real time PCR assays available that can do this. For example, the insertion sequence IS*711 *is considered specific for organisms of the genus *Brucella*. Brucellae can be identified through amplification of this element as demonstrated by Ouahrani-Bettache *et al*. [29] or in the widely used *Abortus-Melitensis-Ovis-Suis *PCR (AMOS-PCR) assay [30]. In addition, there are genus specific real time PCR assays based around the conserved *bcsp*31 target that can fulfil this role [31,32]. However, in light of this observation, we sought to include additional markers that would differentiate *Brucella *from its nearest phylogenetic neighbours. To do this, we looked at the 16S rRNA sequence as the target most commonly used for identification of bacteria to the genus level [33,34]. Alignments of 16S rRNA sequences of *Brucella *spp. with *Ochrobactrum *spp. sequences deposited in GenBank, as well as equivalent sequences for other related α-proteobacteriawere constructed. It should be noted at this point that there are a number of Genbank entries annotated as *Brucella *but which clearly represent *Ochrobactrum*.

**Figure 4:**
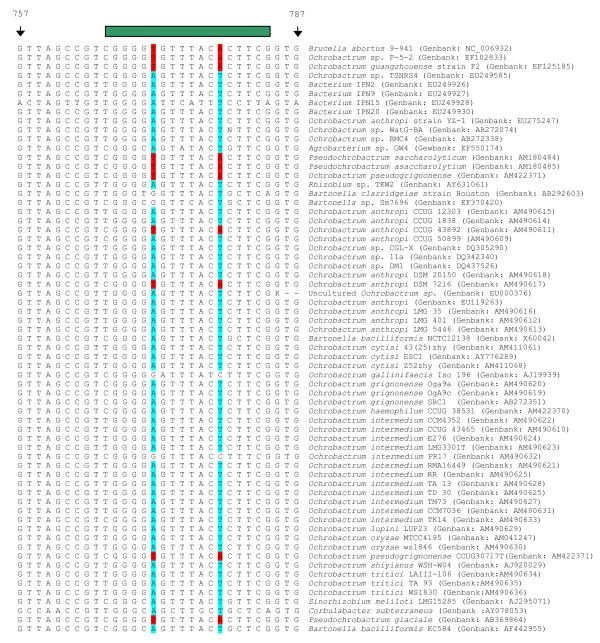
**Alignments of various 16SrRNA sequences around the location of the 16S rRNA_771/778 _probes**. Alignments of fragments of 16S rRNA centred around SNPs at bases 771 and 778 relative to *B. abortus *9–941, used in combination with the 16S rRNA_1055 _SNP to separate *Brucella *from closely related bacteria. The alignment contains 65 sequences taken directly from Genbank and includes many examples of *Ochrobactrum*, the closest phylogentic neighbour of *Brucella *as well as other, less-closely related, members of the α-proteobacteria. In this figure, the targetted *Brucella *specific SNPs are highlighted in red and the non-*Brucella *alternatives in blue. The green bar above both figures represents the location of probe hybridisation. The alignment includes only one *Brucella *sequence as there is 100% identity in the 16SrRNA sequences between all *Brucella *[34]. Using this assay on its own will discriminate most but not all of the non- *Brucella *shown from *Brucella *organisms.

**Figure 5:**
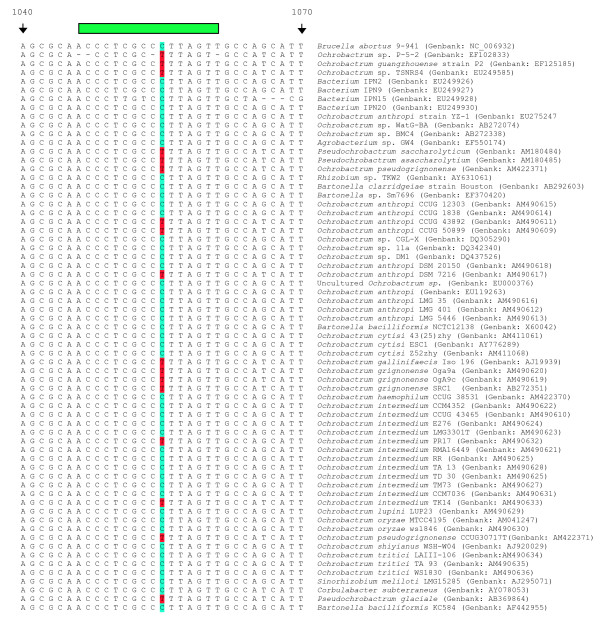
**Alignments of various 16SrRNA sequences around the location of the 16S rRNA_1055 _probes**. Alignments of fragments of 16S rRNA centred around SNP at base 1055 relative to *B. abortus *9–941 showing location of the SNP, used in combination with the 16S rRNA_771/778 _SNPs to separate *Brucella *from closely related bacteria. The alignment contains 65 sequences taken directly from Genbank and includes many examples of *Ochrobactrum*, the closest phylogentic neighbour of *Brucella *as well as other, less-closely related, members of the α-proteobacteria. In this figure, the targetted *Brucella *specific SNP is highlighted in blue and the non-*Brucella *alternatives in red. The green bar above both figures represents the location of probe hybridisation. The alignment includes only one *Brucella *sequence as there is 100% identity in the 16SrRNA sequences between all *Brucella *[34]. Whilst this assay on its own is not as discriminatory as 16S rRNA_771/778 _assay, it crucially distinguishes non-*Brucella *not detected by the afore mentioned assay.

**Figure 6:**
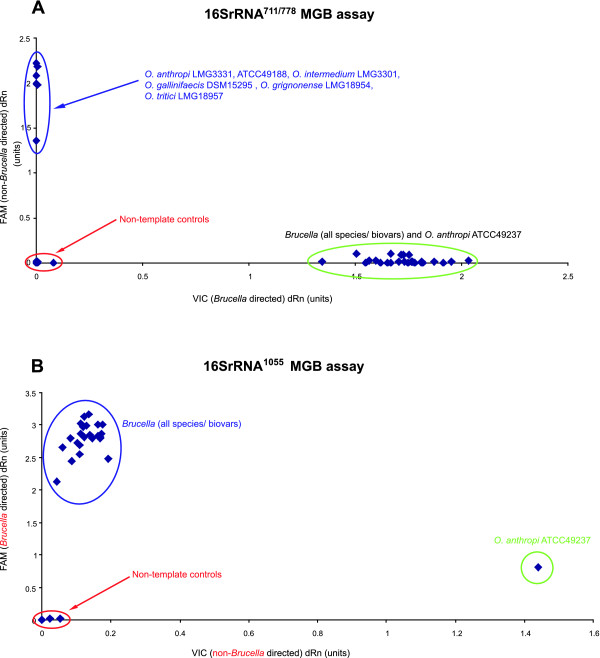
**Discrimination of the *Brucella *genus defining assays**. Application of the two 16S rRNA based probe pairs in distinguishing isolates from the genus *Brucellae *from other α-proteobacteria. a. Results generated by the 16SrRNA_771/778 _probe pair when used against 5 species of *Ochrobactrum *(including three strains of *O. anthropi*) and representatives of all the known *Brucella *species and biovars. This reaction separates *Brucella *isolates from all other except *O. anthropi *ATCC49237. b. Results generated with the application of 16SrRNA_1055 _probe pair illustrating the use of this assay to further separate *O. anthropi *ATCC49237 from *Brucella *isolates.

On this basis three SNPs were identified that when used in conjunction can distinguish *Brucella *from other α-proteobacteria based on the sequences deposited in Genbank currently (December, 2007) (Figures 4 and 5). The three SNPs correspond to positions 771 (16S rRNA_771_), 778 (16S rRNA_778_), and 1055 (16S rRNA_1055_) in the *B. abortus *9–941 ribosomal RNA sequence. MGB probes were designed to discriminate alleles at these three sites, one probing 16S rRNA_771 _and 16S rRNA_778_, the other probing 16S rRNA_1055 _(see Table 1 and Figures 4 and 5). These were tested against all *Brucella *species and biovars as well as the five *Ochrobactrum *type strains previously tested and two additional *Ochrobactrum anthropi *strains (ATCC49188 and ATCC49237). Using this combination of three SNPs manages to distinguish *Brucella *isolates from non-*Brucella *isolates (Figures 6a and 6b).

## Discussion

The aim of this work was to use the robust phylogenetic framework provided by existing MLSA studies to develop a rapid, unambiguous assay for the real-time PCR platform capable of identifying *Brucella *isolates to species level. The approach is based on a series of discrimination assays interrogating SNPs that we have shown to be specific to a particular *Brucella *species. As each individual SNP assay will always give one of two results, species determination is straightforward and unambiguous. Amplification profiles, representing binding of either the VIC- or FAM-labelled probe, are generated in all seven individual discrimination assays. One of the seven individual assays should give a positive reaction with the VIC-labelled probe (or two in the case of *B. canis*), with a reaction with the FAM-labelled probe apparent in all the remaining assays. An assay which did not generate a positive PCR result from each of the seven individual probe pairs would be considered to have failed. This approach avoids any danger of 'false-negatives', where lack of a PCR product could be indicative of PCR inhibition rather than absence of target. Furthermore, although any signals generated by non-*Brucella *organisms in this study were weak, we sought to absolutely ensure the specificity of the assay by identifying three SNPs in 16S rRNA that, when used in conjunction, differentiate *Brucella *from closely related bacteria. Allele discrimination assays based on these three markers were shown to clearly differentiate *Brucella *from closely related α-proteobacteria examined.

As a SNP based speciation assay the approach described here has a number of potential advantages, comparable to those of our primer extension based SNP typing assay [22], when compared to classical biotyping approaches. Both methods overcome the subjectivity associated with biotyping with approaches that are relatively technically straightforward, unambiguous, and have substantial advantages in terms of simplicity and speed. In addition, both techniques reduce the potential exposure to live *Brucella*. This is desirable given the ease of acquisition of *Brucella *in the absence of stringent bio-containment facilities [35,36].

Both assays also have substantial advantages over currently used molecular assays. They can be carried out on crude bacterial lysates bypassing the need for lengthy DNA extraction procedures as required by some molecular typing approaches [20,37] and, as PCR based methods, both techniques should theoretically be applicable directly to field material obviating the need for any culture. In contrast to some extensively used existing assays such as AMOS-PCR [30] these assays are all encompassing identifying all relevant biovars of all species. Of the classically recognised *Brucella *species and biovars only *B. suis *bv 5 will not be positively identified as a particular *Brucella *species by the real-time PCR assay, reflecting its unique phylogenetic position within the group apparently distinct from other *B. suis*. However, like other potentially novel *Brucella *groups, such isolates are positively identified as *Brucella *displaying seven clear non species-specific FAM signals. Both techniques lend themselves to future expansion to include markers for such new groups. For example, after this manuscript was submitted a novel species, *B. microti*, was described. We have already described a SNP specific to this species based on MLSA [9] and probes interrogating this SNP could readily be incorporated into this assay. This capacity for rapid inclusion of newly identified species is a potential advantage over some other molecular assays such as the multiplex PCR assay proposed by Garcia-Yoldi *et al *[38]. Both the primer extension assay and the real-time PCR based assay described here have the advantage of potentially increased sensitivity associated with PCR based techniques. We have shown that the real-time PCR assay described here can detect and differentiate as little as 15 genome equivalents suggesting that it could possibly identify the organism in relevant field material such as foetal tissue or vaginal swabs.

There are, however, substantial advantages in the use of a real-time PCR platform, compared to the primer extension format that led us to investigate and report this alternative approach. The primer extension approach requires a DNA sequencer, while the approach described in this manuscript requires only a real-time PCR machine. Indeed, it could feasibly be undertaken in a simpler format with a conventional thermocycler and a basic fluorescence plate reader. Thus, while the primer extension assay might be appropriate in larger reference laboratories, the technique described in this manuscript is likely to prove far more applicable in routine diagnostics. This is particularly so as real-time PCR machines become more widely available in areas of the world where brucellosis remains a major public and animal health issue. In contrast, the uptake of sequencing based technologies, requiring a substantially greater capital input, is likely to be much slower in these areas. Furthermore, the real-time PCR approach is also technically simpler, faster and cheaper than the primer extension based approach. As amplification and detection is carried out concurrently the real-time assay described here can characterise isolates in around two hours. This compares to the day required for the primer extension based approach that also involves a much less straightforward experimental procedure. Although both primer extension and real-time assays could be further expanded the number of markers that can be included in a single primer extension assay is limited by the need to space markers (ideally by at least 5 bp) between the 13 bp and 88 bp size standards. There is no such limitation with the real-time PCR described here where additional markers can be interrogated simply by the addition of an additional probe pair in another well of a 96 well plate. This will make the latter assay our approach of choice for incorporation of additional markers to type at the sub-species level. Finally, the primer extension assay we described did not include a specific marker for *B. suis*, with this species being recognised by process of elimination (i.e. lack of any of the markers specific for the other classical *Brucella *species). While this was appropriate at the time, the recent description of a novel species of *Brucella *(*B. microti*) indicates the added value of including a specific marker for each species. The inclusion of a marker for *B. suis *in the real-time assay ensures that all species are identified 'positively' by the recognition of a SNP shown, by the extensive population genetic analysis that supports this work, to be specific for the *B. suis/canis *group.

## Conclusion

We have described a simple and unambiguous multiple outcome SNP assay based on a robust phylogenetic framework that can characterise *Brucella *isolates to the species level. The approach is based on a sensitive and rapid real-time PCR platform that will be widely applicable in diagnostic laboratories and is readily expandable as knowledge of the genus increases. Further work will focus on the expansion of the assay to include markers to discriminate live vaccine strains and to type at the sub-species level.

## Methods

### Bacterial isolates

All *Brucella *examined in this study came from the Veterinary Laboratories Agency strain archive. In total, 303 crude extractions from *Brucella *isolates were used to validate the complete assay. These consisted of either boiled cells or crude methanol extractions prepared as described previously [5]. The purified genomic DNA extracts used for sensitivity determination were isolated from *B. abortus *544, *B. melitensis *16 M, *B. suis *1330, *B. ovis *F10/B7/02, *B. canis *79/92, *B. neotomae *65/196, and VLA04/72 (isolated in a porpoise from South West England). The non-*Brucella *used in this work were *Ochrobactrum anthropi *(LMG3301, ATCC49188 and ATCC49237), *O. intermedium *(LMG 3331), *O. gallinifaecis *(DSM15295), *O. grignonense *(LMG18954), and *O. tritici *(LMG18957).

### SNP identification

Identification of SNPs was based on an extension of previously discussed MLSA work [4] to examine twenty-one distinct gene fragments from over 400 isolates of *Brucella*. In addition to the SNPs isolated in four genes *gap*, *omp*25, *trp*E, and *aro*A identified in this previous study [4], SNPs located in three further genes, *fba*A, *prp*E, and a putative oxidoreductase encoding gene (see Table 1) were used in the setting up of this assay. The locations of all SNPs in relation to the sequenced strain, *B. abortus *9–941 is shown in Table 1. In the case of the assay to distinguish *Brucella *spp. from near neighbour organisms 16S rRNA sequence data from a number of different members of the α-proteobacteria group were downloaded from Genbank and aligned with a number of known *Brucella *species using the ClustalW algorithm on the Lasergene 7 Megalign programme (DNAstar, Madison, Wisconsin, USA). Many of these sequences were recently described by Scholz *et al*., [39] who demonstrated separation of *Brucella *from *Ochrobactrum *based on them. Polymorphisms were selected based on the ability to separate *Brucella *sequences as a complete entity from within the remaining α-proteobacteria. In total, three polymorphisms were found that were able to distinguish *Brucella *isolates from their genetic "near neighbours".

### Design of multiple-outcome assay to type Brucella species

The design of this multiple-outcome assay involved the development of seven individual allelic discrimination assays to determine bases present at the seven species defining SNP sites interrogated. Using the MLSA data available, sequences of around 170 bp containing the species defining SNP were edited using the Applied Biosystems File Builder 3.1 software. Probes and primers were designed and provided in a single tube, with the species-specific probe labelled with VIC, and the alternative state probe (reacting with all other species) with FAM. The sequences of both primers and both probes are shown in Table 1. All assays were first checked for species specificity on a small panel of isolates of 27 methanol isolates. All real time PCR reactions were initially run in a final volume of 12.5 μl, comprising of 6.25 μl Taqman universal master mix (Applied Biosystems, Warrington, UK), 900 nM final concentration of each primer, 200 nM final concentration of each probe, and an arbitrary volume of 0.5 μl sample (DNA not quantified). All PCR reactions were run on the Stratagene MX3000P platform (Stratagene, La Jolla, USA) as follows: 1 cycle at 50°C for 2 mins, followed by 1 cycle at 95°C for 10 mins, followed by 40 cycles of 92°C for 15 secs, and 60°C for 1 min. The concentrations of primers and probes were then optimised to maximise discrimination with the least amount of reagents (see Table 2). For all real time reactions involving the validation on 303 isolates and the sensitivity work, the Taqman universal mix was replaced by Taqman Genotyping mix (Applied Biosystems, Warrington, UK) but otherwise, the reactions were the same. For the work involving assay sensitivity genomic DNA, extracted by a phenol/chloroform method [20], was quantified by spectrophotometer (Smartspec Plus, Bio-Rad, Hemel Hempstead, UK). This DNA was then diluted to the required concentration in DNA/RNA free sterile water (Ambion, Huntingdon, UK) prior to replicate testing. For the *Brucella *genus specific MGB dual probe assays, probes and primers were designed around the three SNPs of interest in the 16S rRNA sequence (Table 1). Real time PCR reactions for this part of the study were run as for the validation and sensitivity work for the seven SNP species defining assays.

## Authors' contributions

KKG oversaw all practical work, analyzed the data and drafted the manuscript. MSK contributed to sequencing work that led to the identification of species-specific SNPs. CJS carried out some of the assay development. AMW conceived the study, and participated in its design and coordination, and helped to draft the manuscript. All authors read and approved the final manuscript.
